# Metastasectomy for colorectal pulmonary metastases: a survey among members of the European Society of Thoracic Surgeons

**DOI:** 10.1093/icvts/ivad002

**Published:** 2023-01-09

**Authors:** Martijn van Dorp, Michel Gonzalez, Niccolò Daddi, Hasan F Batirel, Alessandro Brunelli, Wilhelmina H Schreurs

**Affiliations:** Department of Thoracic Surgery, Amsterdam UMC, Vrije Universiteit Amsterdam, Amsterdam, Netherlands; Department of Thoracic Surgery, University Hospital of Lausanne, Lausanne, Switzerland; Department of Thoracic Surgery, Bologna University School of Medicine, Bologna, Italy; Department of Thoracic Surgery, Marmara University School of Medicine, Istanbul, Turkey; Department of Thoracic Surgery, St James’s University, Leeds, UK; Department of Surgery, Northwest Clinics, Alkmaar, Netherlands

**Keywords:** Lung metastases, Metastasectomy, Video-assisted thoracic surgery, Lymphadenectomy, Survey, Colorectal metastases

## Abstract

**OBJECTIVES:**

Surgical management of pulmonary metastases in colorectal cancer patients is a debated topic. There is currently no consensus on this matter, which sparks considerable risk for international practice variation. The European Society of Thoracic Surgeons (ESTS) ran a survey to assess current clinical practices and to determine criteria for resection among ESTS members.

**METHODS:**

All ESTS members were invited to complete an online questionnaire of 38 questions on current practice and management of pulmonary metastases in colorectal cancer patients.

**RESULTS:**

In total, 308 complete responses were received (response rate: 22%) from 62 countries. Most respondents consider that pulmonary metastasectomy for colorectal pulmonary metastases improves disease control (97%) and improves patients’ survival (92%). Invasive mediastinal staging in case of suspicious hilar or mediastinal lymph nodes is indicated (82%). Wedge resection is the preferred type of resection for a peripheral metastasis (87%). Minimally invasive approach is the preferred approach (72%). For a centrally located colorectal pulmonary metastasis, the preferred form of treatment is a minimally invasive anatomical resection (56%). During metastasectomy, 67% of respondents perform mediastinal lymph node sampling or dissection. Routine chemotherapy is rarely or never given following metastasectomy (57% of respondents).

**CONCLUSIONS:**

This survey among the ESTS members underlines the change in practice of pulmonary metastasectomy with an increasing tendency in favour of minimally invasive metastasectomy and surgical resection is preferred over other types of local treatment. Criteria for resectability vary and controversy remains regarding lymph node assessment and the role of adjuvant treatment.

**Figure:**
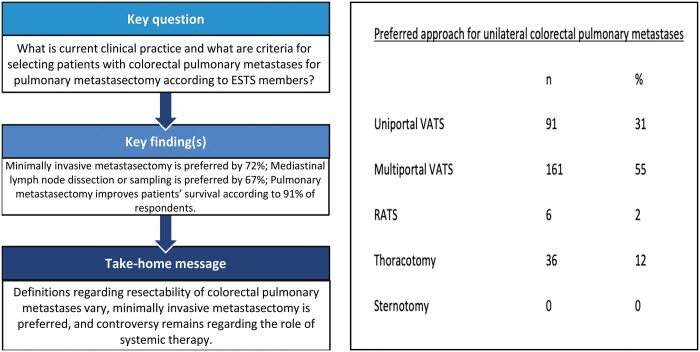

## INTRODUCTION

Pulmonary metastasectomy is widely used as a conventional treatment option for patients with colorectal pulmonary metastases. Surgical resection has historically been proposed to patients who can tolerate surgery and in whom all pulmonary metastases are amenable to resection [1]. Metastasectomy offers the highest local control in comparison to other local treatment options [2]. However, selection of patients for any form of local therapy is crucial and great variations exist among the implemented selection criteria.

The European Society of Thoracic Surgeons (ESTS) has performed a previous survey on pulmonary metastasectomy in 2008 [3] with a response rate of 29.6%, which was not specified to one specific primary tumour type. Although around 50% of resected pulmonary metastases are of colorectal origin [4]. This survey demonstrated that palpation of the lung was deemed mandatory by 65% of respondents, and that 60% of surgeons considered a thoracoscopic approach only an option for diagnostic purposes. In the last decade, minimally invasive approaches for pulmonary metastases have gained acceptance and radiological imaging has considerably improved, as shown by the steadily increasing minimally invasive metastasectomy rates in the ESTS database [5] and several other published national registries [4, 6, 7]. Yet, these published registries also revealed that there are still considerable variations regarding the indication for metastasectomy, the necessity of lymph node dissection and the role of systemic treatment.

The main goal of pulmonary metastasectomy remains to achieve a complete resection of the metastases while preserving as much pulmonary parenchyma as possible. Several guidelines have been published on the treatment of metastatic colorectal cancer [8–10]. However, these guidelines fail to define the role of metastasectomy for pulmonary metastases in colorectal cancer patients. Several consensus documents [11–13] have been published to improve patient selection, but they are mainly based on expert opinions due to the lack of high-quality clinical research. In addition, surgical resection is questioned by the recent publication of the PulMiCC trial [14], which reports that selected colorectal cancer patients have better survival without pulmonary metastasectomy than previously assumed.

To assess the current clinical practices and to determine criteria for resection among thoracic surgeons regarding pulmonary metastasectomy for colorectal metastases, the ESTS decided to survey its members. The aim was to update and document the changes in practice during the last decade for pulmonary metastasectomy in colorectal cancer patients.

## MATERIALS AND METHODS

### Ethical statement

All ESTS members (1433 as of June 2021) received an e-mail informing them about the survey. Members were invited to complete the questionnaire from June 2021 through November 2021 using a commercially available format (www.surveymonkey.com). This study was approved by the ESTS council. During this 6-month period, 3 reminders were sent via e-mail to the ESTS members to boost responses prior to study closure. A social media campaign was also implemented to disseminate the survey (Twitter and LinkedIn) and to improve the response rate. All responses were voluntary and anonymous.

### Survey design

The questionnaire contained 38 questions, divided into 6 sections. In the first 7 questions, demographic data of the respondents were collected, which included the level of practice, years in practice, country of practice, number of metastasectomy cases per year and the percentage of metastasectomy cases in the general clinical volume. The second section contained 8 questions and was based on preoperative evaluation. This included the role of, and contra-indications for surgery, review at multidisciplinary tumour board, use of positron emission tomography–computed tomography (PET-CT) and carcinoembryonic antigen measurements, the need for preoperative invasive lymph node assessment and tissue biopsy. The third section contained 6 questions on the surgical approach and included the preferred (minimally invasive or open) approach and the strategy for bilateral metastases management. Five questions followed on the extent of resection, including central metastases (requiring an anatomical resection), the need for pneumonectomy and treatment of inoperable patients. Five questions then covered lymph node assessment, including lymph node sampling or dissection, with or without suspect hilar or mediastinal lymph nodes. And finally, 6 questions were asked on postoperative management, including the role of chemotherapy, biomarkers and follow-up. One additional open-ended question was presented to allow for comments and considerations. In formulating specific questions, we synchronized some questions to the previous ESTS survey on pulmonary metastasectomy to allow for comparison [3]. The answers were carefully worded to avoid ambiguous and uninformative answers. All questions and possible answers are provided as Supplementary Material, Appendix S1.

### Statistical analyses

Only fully complete questionnaires were considered for analysis. The results from trainee responses were not included in this analysis. Data are presented as numbers and percentages. Percentages are rounded to one decimal place. Categorical variables were tested using the Pearson’s χ^2^ test or Fisher’s exact test as appropriate. The analysis was performed using IBM SPSS statistics, version 28.0.

## RESULTS

**Figure 1:**
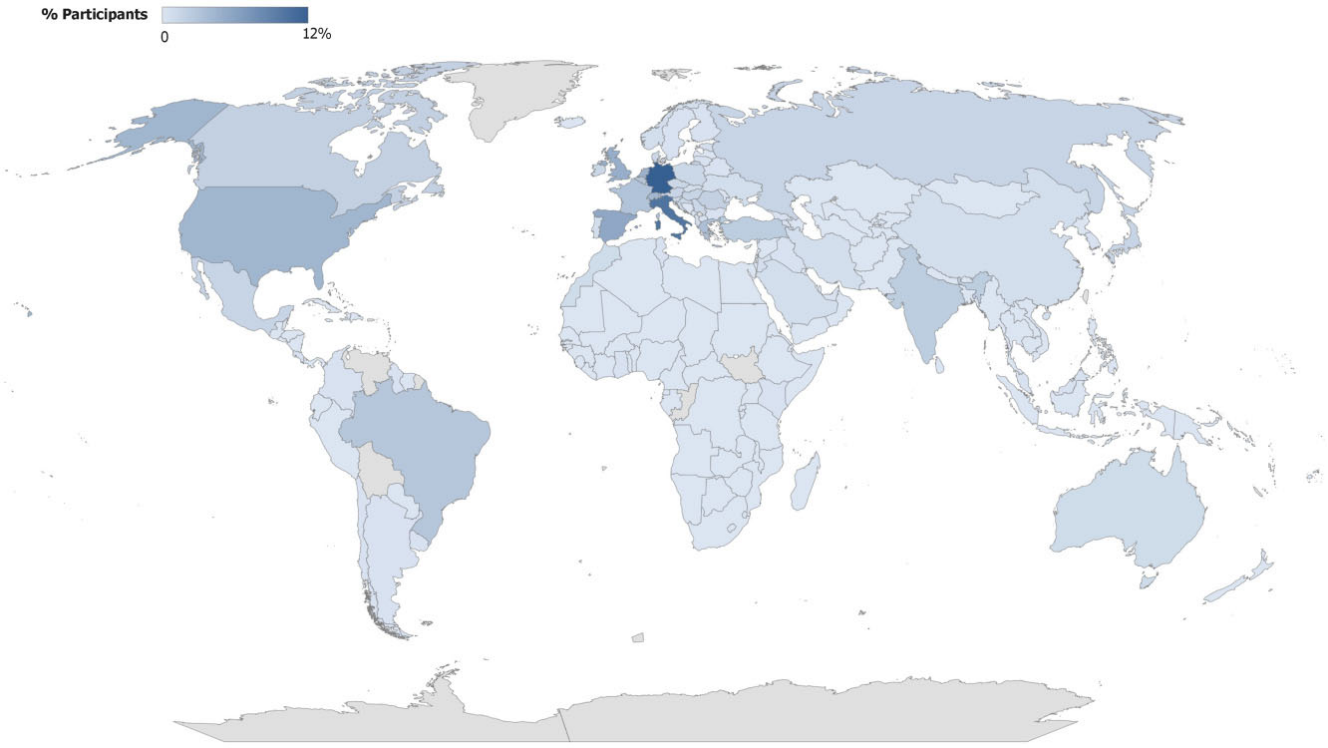
Geographic distribution of responding ESTS members.

A total of 321 responses (response rate 22.4%) was received and all questionnaires were complete (completeness rate 100%). Thirteen complete questionnaires were received from trainees and excluded from analysis. The geographic distribution of the 308 ESTS members from 62 countries is presented in Fig. 1.

### Respondents

One hundred and sixty-two (52.8%) participants work in an academic hospital, 125 (40.7%) work in a public hospital and 20 (6.5%) in a private hospital. One hundred and ninety-six (63.6%) respondents are consultant surgeons, 83 (26.9%) are professors, 22 (7.1%) fellows and 7 (2.3%) retired. Thirty-five percent of respondents have <10 years of experience as thoracic surgeons, 55% have 10–30 years of experience and 10% have more than 30 years of experience.

In total, 98.7% of respondents perform pulmonary metastasectomy in their clinical practice with a variable number of pulmonary metastasectomy cases (median 22, interquartile range 15–40) per hospital per year. However, pulmonary metastasectomy represents a minor proportion of the general clinical volume: 0–10% for 61.6% of respondents and 11–25% for 32.5% of respondents.

### Preoperative evaluation

Regarding the goal of pulmonary metastasectomy for colorectal pulmonary metastases, 91.6% of respondents consider that the reason for performing pulmonary metastasectomy is to improve survival, 96.6% to improve disease control and 63.6% to improve patients’ quality of life. In addition, 30.3% of the participants claim that there is unproven benefit, whereas 3.8% consider pulmonary metastasectomy obsolete.

In total, 90.0% of respondents always or usually review pulmonary metastasectomy cases in a multidisciplinary tumour board. Furthermore, 81.5% of participants always or usually perform preoperative PET-CT and 78.2% always or usually measure the preoperative carcinoembryonic antigen level prior to pulmonary metastasectomy. However, tissue biopsy of colorectal pulmonary metastases is rarely or never performed preoperatively according to 68.6% of participants.

Invasive preoperative mediastinal lymph node staging should always be performed prior to pulmonary metastasectomy according to 19 participants (6.1%) and 35 participants (11.4%) indicate that invasive mediastinal lymph node staging should never be performed preoperatively. The majority prefer to only select patients with hilar or mediastinal enlarged or PET-avid lymph nodes for invasive staging (82.4%). A preference for endosonography (endobronchial ultrasound or endoscopic ultrasound) is expressed by 72.0% of respondents for preoperative invasive mediastinal lymph node staging, followed by cervical mediastinoscopy (11.7%) and transcervical extended mediastinal lymphadenectomy (0.3%).

### Surgical approach

The preferred surgical approach for pulmonary metastasectomy for colorectal metastases is a minimally invasive approach [video-assisted thoracic surgery (VATS) or robot-assisted thoracic surgery] without bimanual palpation of the lung and without rib spreading according to 72.4% of respondents, whereas 27.6% still prefer an open approach with bimanual palpation. Surgeons with more than 20 years of experience are significantly more inclined to perform an open resection compared to surgeons with <20 years of experience (34.9% vs 22.7%, *P* = 0.003).

A minimally invasive approach is preferred for solitary metastases (96.7%), peripheral metastases (94.8%), bilateral metastases (80.3%), patients with a poor performance score (85.9%) or with advanced age (84.1%). However, an open approach is recommended as the preferred approach for multiple metastases (55.4%), central metastases (70.6%), large metastases (63.2%) or to avoid unnecessary resection of lung parenchyma (65.6%). In case of bilateral pulmonary metastases, the preferred approach is by VATS in 81.2% (22.0% for single stage thoracoscopy and 59.2% for staged thoracoscopy) (Table 2). The preferred time-interval for staged resections is 1–4 weeks (54%) followed by 5–8 weeks (44.7%).

### Extent of resection and surgical technique

For a peripheral metastasis, the most common form of resection is stapled wedge resection (88.1%), followed by anatomical segmentectomy (6.6%) and laser resection (5.2%). For a centrally located colorectal pulmonary metastasis, the preferred form of resection is a minimally invasive anatomical resection (segmentectomy or lobectomy) (55.5%), followed by open resection (38.5%), stereotactic ablative radiotherapy (5.0%) and radiofrequency ablation or microwave ablation (1.0%).

The absolute maximum number of colorectal pulmonary metastases that is considered resectable is 1–2 (5%), 3–4 (35%), 5–7 (23%) and more than 8 metastases (37%). For inoperable patients, stereotactic ablative radiotherapy (68.1%) or radiofrequency ablation/microwave ablation (18.0%) are the preferred options for local treatment. However, 13.8% of respondents select not to perform any local therapy for inoperable patients. Pneumonectomy is considered a valid treatment option to achieve a complete resection of colorectal pulmonary metastases (in highly selected patients) in 66% of responses.

### Lymph node assessment

Pathologically proven mediastinal lymph node metastases of colorectal pulmonary metastases are considered an absolute contraindication for surgery by 70% of respondents (Table 1). During pulmonary metastasectomy, 67.1% of surgeons perform lymph node assessment by means of mediastinal lymph node sampling (45.0%) or mediastinal lymph node dissection (22.1%). More surgeons are inclined to perform lymph node assessment (by means of sampling or dissection) for central metastases compared to peripheral metastases (79.3% vs 56.4%, *P* < 0.001). Furthermore, more surgeons perform a radical lymph node dissection for central metastases than for peripheral metastases (39.8% vs 16.1%, *P* < 0.001). Surgeons who prefer a minimally invasive approach perform intraoperative lymph node assessment less frequently than surgeons who prefer an open resection (59.4% vs 88.1%, *P* < 0.001). In Table 3, the preferred approach for suspect hilar or mediastinal lymph node metastases is shown.

**Table 1:** Absolute contraindications in the current survey and the previous survey [3]

	Current survey, *n* (%)	Previous survey, *n* (%)
Multiple (>1) colorectal pulmonary metastases	17 (6)	0 (0)
Bilateral colorectal pulmonary metastases	25 (8)	2 (1)
Previous colorectal pulmonary metastases	18 (6)	1 (1)
Concurrent colorectal liver metastases	71 (24)	49 (34)
Poor performance status (Karnofsky score <50%)	258 (87)	56 (38)
Poor lung function (FEV1 or DLCO <40%)	208 (70)	37 (25)
Pathologically proven mediastinal lymph nodes	208 (70)	94 (64)
Unresectable primary malignancy	295 (96)	134 (92)

FEV: forced expiratory value; DLCO: diffusing capacity for carbon monoxide.

**Table 2:** Surgical approach for unilateral or bilateral colorectal pulmonary metastases

	Preferred approach for unilateral colorectal pulmonary metastases	
	*n*	%
Uniportal VATS	91	31
Bi-portal VATS	80	27
Three-portal VATS	81	28
RATS	6	2
Thoracotomy	36	12
Sternotomy	0	0
	Preferred approach for bilateral colorectal pulmonary metastases	
Bilateral simultaneous VATS/RATS	63	22
Bilateral staged VATS/RATS	170	59
Sternotomy	3	1
Clamshell	2	1
Bilateral simultaneous thoracotomy	6	2
Bilateral staged thoracotomy	43	15

VATS: video-assisted thoracic surgery; RATS: robot-assisted thoracic surgery.

**Table 3:** Approach for simultaneous hilar or mediastinal lymph node metastases

	Radiologically suspect hilar (N1) lymph nodes	
	*N*	%
Endosonography (EBUS or EUS)	130	43
Cervical mediastinoscopy	9	3
Pulmonary metastasectomy with mediastinal lymph node sampling	60	20
Pulmonary metastasectomy with mediastinal lymph node dissection	97	31
Watch-and-wait	9	3
	Radiologically suspect mediastinal (N2) lymph nodes	
Endosonography (EBUS or EUS)	178	60
Cervical mediastinoscopy	32	11
Pulmonary metastasectomy with mediastinal lymph node sampling	16	5
Pulmonary metastasectomy with mediastinal lymph node dissection	57	19
Watch-and-wait	16	5

EBUS: endobronchial ultrasound; EUS: endoscopic ultrasound.

### Chemotherapy and follow-up

Patients ‘always or usually’ receive preoperative chemotherapy according to 41.6% of respondents and 58.4% report that patients ‘rarely or never’ receive preoperative chemotherapy. With regards to adjuvant chemotherapy, 53.9% usually or always recommend adjuvant chemotherapy following complete (R0) resection of colorectal pulmonary metastases and this percentage increases to 92.9% of participants that recommend adjuvant chemotherapy following pulmonary metastasectomy for colorectal pulmonary metastases with involved mediastinal lymph nodes.

Biomarkers (KRAS, NRAS and BRAF) are usually or always determined according to 74.2% of respondents. Follow-up is performed with chest computed tomography scan (96.7%), PET-CT (36.7%) and carcinoembryonic antigen marker determination (84.3%). The most popular interval for radiological imaging is 3–6 months (97.7%).

## DISCUSSION

Pulmonary metastasectomy for colorectal pulmonary metastases is widely performed and 98.7% of respondents perform pulmonary metastasectomy in their clinical practice. However, for most surgeons, it accounts for a minor part of the surgical volume. Most respondents consider that pulmonary metastasectomy for colorectal pulmonary metastases improves disease control (94%) or improves patients’ survival (91%).

When considering the evolution of pulmonary metastasectomy over the last decade, an increase in the use of minimally invasive techniques can be observed as demonstrated in Table 4. The ESTS database on metastasectomy revealed a nearly yearly increase in the percentage of VATS procedures from 2007 to 2019, from 15% in 2007 to 56% in 2019 [5]. The Dutch Lung Cancer Audit analysed 2090 metastasectomy cases from 2012 to 2017 and found that 74% of procedures were performed by VATS [4]. Both the ESTS database and the Dutch database describe a low conversion rate of 2.1% and 3.8%, respectively. The previous ESTS survey on pulmonary metastasectomy [3] reported that 65% of responding surgeons deemed palpation of the lung mandatory and that VATS was the preferred approach for unilateral metastases in 22%. In the current survey, a striking 86% of respondents prefer VATS for unilateral metastases. The previous survey stated that 20% preferred VATS for bilateral metastases with 8% for single-stage resection and 12% for staged resection. In the current survey, a marked difference was observed, and 81% of respondents preferred VATS for bilateral metastases, with 22% for single-stage resection and 59% for staged resection.

**Table 4:** Large registries on pulmonary metastasectomy

Author	Registry	Time frame	No patients	Minimally invasive metastasectomy (%)	Lymph node sampling or dissection (%)
Pastorino *et al.* 1997 [15]	International registry of lung metastases (IRLM)	1991–1995	5206	2	ND
Casiraghi *et al.* 2011 [16]	Milan	1998–2008	575	3	65
Hernández *et al.* 2016 [6]	Spanish Group of Lung Metastases (GECMP)	2008–2010	522	18	48
Okumura *et al.* 2017 [7]	Metastatic lung tumour study group of Japan	1999–2014	1047	38	58
Gonzalez *et al.* 2021 [5]	ESTS database	2007–2019	8868	37	57
van Dorp *et al.* 2020 [4]	Dutch lung cancer audit (DLCA)	2012–2019	2090	74	12

NP: not determined.

A non-anatomical resection is the preferred form of resection, with 88% of respondents favouring a stapled wedge resection for a peripheral metastasis. This has not changed over time: in the previous survey, 89% of respondents preferred a stapled wedge resection. The ESTS database [5], the International registry [15] and the Dutch national database [4] all describe stapled wedge resection as the most frequently performed procedure, with 60%, 67% and 70% of cases, respectively. The tendency towards performing intraoperative mediastinal lymph node assessment (sampling or dissection) has not changed over time either, with 68% of respondents performing intraoperative lymph node assessment in the previous survey and 67% in the current survey. However, significantly more respondents are willing to perform lymph node assessment for central metastases compared to peripheral metastases (79% vs 56%). The Society of Thoracic Surgeons expert consensus proposed that lymph node assessment during metastasectomy should be considered to predict survival [12]. The ESTS database described that lymph node dissection or sampling was realized in 57% and noted that only 36% of patients underwent lymph node assessment when surgery was carried out by VATS, in comparison to 70% when an open procedure was performed. This was also observed in the results of the survey, where significantly more surgeons perform lymph node assessment during an open approach compared to a minimally invasive approach (88% vs 59%). Recent systematic review on pulmonary metastasectomy with lymphadenectomy objectified a detrimental effect of mediastinal lymph node involvement with a 5-year survival rate of 10.9% for colorectal cancer patients [17]. Variable lymph node assessments rates are seen in several national databases (Table 4). In a cross-sectional survey of international experts, intraoperative lymph node assessment was only recommended by 50% of experts [18]. Currently, a Danish randomized controlled trial assess the impact of pulmonary metastasectomy with lymphadenectomy for colorectal pulmonary metastases [19].

Seventy percent of respondents stated that pathologically proven mediastinal lymph node metastases of colorectal pulmonary metastases are considered an absolute contra-indication. Overall, 82% of participants preferred to select patients with suspect hilar or mediastinal lymph nodes on preoperative imaging for invasive lymph node staging. Similarly, 72% prefer endosonography (endobronchial ultrasound or endoscopic ultrasound) over surgical staging for preoperative invasive mediastinal lymph node staging. The possible need for preoperative invasive mediastinal lymph node is mostly determined by preoperative imaging. Patients with suspect hilar lymph nodes are considered candidates for preoperative invasive lymph node staging by 46% of respondents. Patients with suspect mediastinal lymph nodes are considered candidates for preoperative invasive lymph node staging by 71%. For both groups, a watch-and-wait approach was preferred by 3% and 5%, respectively. Based on retrospective data, the incidence of lymph node metastases during metastasectomy for colorectal pulmonary metastases is 19.1% and the incidence of mediastinal lymph node metastases is 10.8% [17]. In the cross-sectional survey of international experts, preoperative lymph node assessment was recommended by 60% of experts for enlarged or PET-avid lymph nodes [18].

Treatment of bilateral colorectal pulmonary metastases remains a controversial issue and 6% of respondents even state that bilateral metastases are an absolute contraindication. Staged procedures are preferred over a single-stage procedure (74% vs 24%). This is independent of the approach (minimally invasive or open). Interestingly, in the previous survey from 2008, the preferred approach for clinical bilateral disease was a bilateral staged thoracotomy (66%), followed by a single-stage bilateral thoracotomy (19%). In the current survey, 81% of surgeons select a minimally invasive approach for clinical bilateral disease. Only 4% of participants are inclined to perform either a sternotomy, clamshell procedure or bilateral single-stage thoracotomy for bilateral metastases. This is in line with the increasing tendency towards minimally invasive metastasectomy. However, an increasing tendency towards minimally invasive metastasectomy should not be confused with a less aggressive approach regarding the surgical treatment of colorectal pulmonary metastases. Sixty percent of respondents consider more than 5 metastases resectable and 66% consider pneumonectomy a valid treatment option to achieve a complete resection of colorectal pulmonary metastases (in highly selected patients). In a survey performed by the members of the Society for Cardiothoracic Surgery in Great Britain and Ireland, 85% of respondents stated that bilateral colorectal metastases were considered an indication for metastasectomy [20].

Several variables are considered absolute contra-indications to surgery by a higher percentage of respondents in the current survey than in the previous survey as noted in Table 1. These include multiple metastases, bilateral metastases, previous pulmonary metastases, poor performance score, poor lung function, mediastinal lymph node involvement and presence of an unresectable primary tumour. The RAS and BRAF biomarkers are generally determined by 74% of surgeons. KRAS mutations are independent prognostic factors for survival [21] but are also associated with a shorter time to the development of lung metastases [22]. The role of neoadjuvant and adjuvant chemotherapy is considered controversial. The indication for chemotherapy is determined by a medical oncologist and discussed in the multidisciplinary tumour board. Therefore, these raw percentages only reflect patterns of care. A recent meta-analysis revealed that adjuvant chemotherapy after resection of colorectal pulmonary metastasis did not improve overall survival and did not show improved survival for the subgroup with hilar or mediastinal lymph node involvement [23]. And a propensity-matched analysis did also not reveal a survival benefit for adjuvant chemotherapy after pulmonary metastasectomy [24]. Despite these results, 93% of respondents recommend adjuvant chemotherapy following pulmonary metastasectomy for colorectal pulmonary metastases with mediastinal lymph node involvement.

The total amount of completed questionnaires was more than double compared to 2008 (308 vs 146). However, due to the increase in ESTS members over time, the percentage of members who responded was higher in the previous survey (29.6% in 2008 vs 22.4% in 2021). The data in this survey suffers from nonresponse bias because surgeons who perform metastasectomy are more likely to participate. It is possible that the outcome of the respondents is not representative for all ESTS members. Demand characteristics bias can affect the outcome, given that participants of a survey can change their opinion because of taking part in a study. Sample size was not determined in advance. The multiple-choice answers were formatted to avoid ambiguous and uninformative answers. Answering ‘sometimes’ as a multiple-choice option was not possible to allow for more definitive answers. Not all known prognostic factors can be considered when formulating specific questions, factors like disease-free interval, patient performance status and previous treatment of colorectal cancer are difficult to incorporate in a questionnaire. The findings of the study do not serve as a recommendation for the treatment of colorectal pulmonary metastases, they merely reflect the current clinical practices and criteria for metastasectomy among ESTS members.

This survey among the ESTS members and its comparison to the prior survey underlines the change in practice of pulmonary metastasectomy with an increasing tendency in favour of minimally invasive metastasectomy for colorectal pulmonary metastases. Criteria for resectability vary, and controversy remains regarding lymph node assessment during metastasectomy and the role of adjuvant treatment.

## Supplementary Material

ivad002_Supplementary_Data

## Data Availability

All relevant data are within the manuscript and its supporting information files.

## ABBREVIATIONS

ESTS: European Society of Thoracic Surgeons
PET-CT: Positron emission tomography–computed tomography
VATS: Video-assisted thoracic surgery
