# Supplementary material for: Metastasectomy for colorectal pulmonary metastases: a survey among members of the European Society of Thoracic Surgeons
Source: Interdiscip Cardiovasc Thorac Surg. 2023 Jan 9;36(2):ivad002. doi: 10.1093/icvts/ivad002 (PMC9932002; doi:10.1093/icvts/ivad002)
Supplement: ivad002_Supplementary_Data [file ivad002_supplementary_data.pdf]

ESTS questionnaire on Pulmonary Metastasectomy for Colorectal Pulmonary Metastases.

Treatment of colorectal pulmonary metastases by means of surgical resection remains a topic of debate. Despite the available literature, there is considerable risk for national and international practice variation.

We would like to ask the ESTS members to participate in this online survey to investigates the current clinical practice among European Society of Thoracic Surgeon (ESTS) members.

The survey is anonymous and will take 7-8 minutes to complete.

Questionnaire

Responders

1. Type of practice?

- Academic
- Public
- Private

2. Level of practice?

- Trainee
- Fellow
- Consultant surgeon
- Professor
- Retired / not currently in practice

3. Years in practice as a thoracic surgeon?

- …

4. Country of practice?

- …

5. Do you perform pulmonary metastasectomy for colorectal pulmonary metastases in your clinical practice?

- Yes
- No

6. How many pulmonary metastasecomy cases do you approximately perform in your hospital per year?

- …

7. As a proportion of your clinical volume, pulmonary metastasectomy accounts for approximately what percentage of your daily practice?

- 0-10%
- 10-25%
- 25-50%
- >50%

Preoperative workup for colorectal pulmonary metastases

8. How do you generally consider the role of pulmonary metastasectomy for colorectal pulmonary metastases?

- Improve survival (Y/N)
- Improve disease controle (Y/N)
- Unproven benefit (Y/N)
- Quality of life (Y/N)
- Obsolete (Y/N)

9. Do you consider the following as <u>absolute contraindication</u> to pulmonary metastasectomy for colorectal pulmonary metastases?

- Multiple (>1) colorectal pulmonary metastases (Y/N)
- Bilateral colorectal pulmonary metastases (Y/N)
- Previous colorectal pulmonary metastases (Y/N)
- Synchronous colorectal pulmonary metastases (Y/N)
- Concurrent colorectal liver metastases (Y/N)
- Poor performance status (Karnofsky score <50%) (Y/N)
- Poor lung function (FEV1 or DLCO <40% of predicted) (Y/N)
- Pathologically proven mediastinal lymph nodes (Y/N)
- Unresectable primary malignancy (Y/N)
- Requiring extended resection to other structures (chest wall, diaphragm, vena cava) (Y/N)

10. Do you review pulmonary metastasecomy cases in a multidisciplinary tumor board?

- Always, every case
- Usually
- Rarely
- Never

11. Do you perform a PET-CT prior to pulmonary metastasectomy for colorectal pulmonary metastases?

- Always, every case
- Usually
- Rarely
- Never

12. Do you routinely measure CEA levels prior to pulmonary metastasectomy for colorectal pulmonary metastases?

- Always, every case
- Usually
- Rarely
- Never

13. Would you recommend preoperative invasive lymph node assessment prior to pulmonary metastasectomy for colorectal pulmonary metastases?

- Always, every case
- Only when hilar/mediastinal lymph nodes are enlarged or PET-avid
- Never

14. How would you prefer to assess mediastinal lymph nodes prior to pulmonary metastasectomy for colorectal pulmonary metastases?

- Endosonography (EBUS or EUS)
- Cervical mediastinoscopy
- TEMLA
- I never perform preoperative lymph node assessment for colorectal pulmonary metastases

15. Would you recommend tissue biopsy of colorectal pulmonary metastases prior to surgical resection?

- Always, every case
- Usually
- Rarely
- Never

Surgical approach for colorectal pulmonary metastases

16. What is your preferred approach to pulmonary metastasectomy for colorectal pulmonary metastases?

- Open approach with bimanual palpation
- Minimal invasive without bimanual palpation

17. In which situation would you recommend thoracoscopy

- Solitary metastasis (Y/N)
- Peripheral metastases (Y/N)
- Bilateral metastases (Y/N)
- Poor performance status (Y/N)
- Advanced age (Y/N)
- Only for diagnosis (Y/N)

18. In which situation would you recommend an open approach

- Multiple metastases (Y/N)
- Central metastases (Y/N)
- Bilateral metastases (Y/N)
- To avoid unnecessary lung resection (Y/N)
- Large size pulmonary metastases (Y/N)
- I always perform thoracotomy (Y/N)

19. What would you recommend as the preferred approach to resecting unilateral colorectal pulmonary metastases?

- (u)VATS
- RATS
- Thoracotomy
- Sternotomy
- Other

20. What would you recommend as the preferred approach to resecting bilateral colorectal pulmonary metastases?

- Bilateral single-stage thoracoscopy
- Bilateral staged thoracoscopy
- Sternotomy
- Clamshell
- Bilateral single-stage thoracotomy
- Bilateral staged thoracotomy
- Other

21. In the case of bilateral staged pulmonary metastasectomy, what time interval do you consider between the 2 operations?

- 1-4 weeks
- 4-8 weeks
- >8 weeks

Extend of resection and surgical technique

22. Which type of resection do you prefer for a solitary peripheral colorectal pulmonary metastasis, if all options are possible

- Stapled wedge resection
- Anatomical segmentectomy
- Lobectomy
- Laser resection

23. What type of treatment do you prefer for a centrally located colorectal pulmonary metastasis in an operable patient?

- Open resection to avoid unnecessary lung resection
- VATS anatomical resection (segment or lobe) to avoid morbidity related to thoracotomy
- Stereotactic radiotherapy
- Radiofrequency / microwave ablation
- Other

24. What type of treatment do you prefer for colorectal pulmonary metastases in an inoperable patient?

- Stereotactic radiotherapy
- Radiofrequency / microwave ablation
- No local treatment

25. Would you perform a pneumonectomy to achieve a complete resection of colorectal pulmonary metastases?

- Yes, in any cases if necessary
- Only in a highly selected fit patient
- No

26. What is the absolute maximum number of colorectal pulmonary metastases that you are willing resect, considering that complete resection is possible

- 1 to 2
- 3 to 4
- 5 to 7
- 8 or more

Lymph node assessment
27. What type of lymph node assessment do you routinely perform during pulmonary metastasectomy for colorectal pulmonary metastases?

- No lymph node sampling/dissection
- Mediastinal lymph node sampling
- Mediastinal lymph node dissection

28. What type of lymph node assessment do you routinely perform during pulmonary metastasectomy for peripheral colorectal pulmonary metastases?

- No lymph node sampling/dissection
- Mediastinal lymph node sampling
- Mediastinal lymph node dissection

29. What type of lymph node assessment do you routinely perform during pulmonary metastasectomy for central (requiring an anatomical resection) colorectal pulmonary metastases?

- No lymph node sampling/dissection
- Mediastinal lymph node sampling
- Mediastinal lymph node dissection

30. What type of approach do you prefer for colorectal pulmonary metastases with suspect hilar (N1) lymph nodes on preoperative imaging?

- Endosonography (EBUS or EUS)
- Cervical mediastinoscopy
- Pulmonary metastasectomy with mediastinal lymph node sampling
- Pulmonary metastasectomy with mediastinal lymph node dissection
- Watch-and-wait
- Other

31. What type of approach do you prefer for colorectal pulmonary metastases with suspect mediastinal (N2) lymph nodes on preoperative imaging?

- Endosonography (EBUS or EUS)
- Cervical mediastinoscopy
- Pulmonary metastasectomy with mediastinal lymph node sampling
- Pulmonary metastasectomy with mediastinal lymph node dissection
- Watch-and-wait
- Other

Chemotherapy

32. Do you routinely administer preoperative chemotherapy before pulmonary metastasectomy for resectable colorectal pulmonary metastases?

- Always, every case
- Usually
- Rarely
- Never

33. Would you recommend adjuvant chemotherapy after complete resection of colorectal pulmonary metastases?

- Always, every case
- Usually
- Rarely
- Never

34. Would you recommend adjuvant chemotherapy after complete resection of colorectal pulmonary metastases with positive mediastinal lymph nodes?

- Always, every case
- Usually
- Rarely
- Never

35. Do you routinely analyze biomarkers (RAS status, BRAF, etc) on pulmonary specimen?

- Always, every case
- Usually
- Rarely
- Never

36. What are your standard investigations during follow-up after pulmonary metastasectomy?

- Chest CT scan (Y/N)
- PET-CT scan (Y/N)
- CEA (Y/N)

37. After pulmonary metastasectomy, at what time intervals do you recommend radiological imaging?

- 3 months
- 6 months
- 12 months
- 2 years
- I do not recommend radiological follow-up
- None of the above

38. Feel free to add any comments/considerations to this questionnaire

- …
